# Mixed Photon and Carbon-Ion Beam Radiotherapy in the Management of Non-Metastatic Nasopharyngeal Carcinoma

**DOI:** 10.3389/fonc.2021.653050

**Published:** 2021-07-23

**Authors:** Jiyi Hu, Qingting Huang, Jing Gao, Weixu Hu, Jing Yang, Xiyin Guan, Xianxin Qiu, Wenna Zhang, Lin Kong, Jiade J. Lu

**Affiliations:** ^1^ Department of Radiation Oncology, Shanghai Proton and Heavy Ion Center, Shanghai, China; ^2^ Shanghai Engineering Research Center of Proton and Heavy Ion Radiation Therapy, Shanghai, China; ^3^ Shanghai Key Laboratory of Radiation Oncology (20dz2261000), Shanghai, China; ^4^ Department of Radiation Oncology, Shanghai Proton and Heavy Ion Center, Fudan University Cancer Hospital, Shanghai, China

**Keywords:** nasopharyngeal carcinoma, intensity modulated radiation therapy, carbon ion radiation therapy (CIRT), survival, disease control, toxicity

## Abstract

**Background:**

Carbon-ion radiotherapy (CIRT) may further increase the therapeutic ratio for patients with newly diagnosed nasopharyngeal carcinoma (NPC). The purpose of the current study is to examine the effectiveness and toxicity profile of photon-based intensity-modulated radiotherapy (IMRT) plus CIRT boost in a relatively large cohort of NPC patients.

**Methods:**

In the current study, non-metastatic NPC patients treated with IMRT plus CIRT boost at Shanghai Proton and Heavy Ion Center between June, 2015 and June, 2018 were included. Overall survival (OS), progression-free survival (PFS), local control, regional control, and distant control were calculated with Kaplan–Meier method. Acute and late toxicities were graded using CTCAE 4.03.

**Results:**

A total of 69 patients were included in the analysis. Among those, 74% of the patients had locoregionally advanced (stage III/IV) disease, and 92.8% had cervical lymphadenopathy. With a median follow-up of 31.9 months, the 3-year OS, PFS, local control, regional control, and distant control rates were 94.9, 85.2, 96.9, 98.4, and 89.7%, respectively. Mixed treatment of IMRT with CIRT boost was well tolerated. Severe acute toxicities induced by radiation therapy were observed in only two patients (dermatitis). No severe radiation-induced late toxicity was observed at the time of analysis. Univariable analysis showed N2/3 disease was correlated with an inferior distant control (*p* = 0.040).

**Conclusion:**

Mixed treatment of IMRT plus CIRT boost provides an excellent disease control and a favorable toxicity profile for patients with non-metastatic NPC. Further follow-up is necessary to evaluate the long-term survivals and toxicities more accurately.

## Introduction

Nasopharyngeal carcinoma (NPC) is one of the most commonly diagnosed head and neck cancers in Southeast Asia. Radiation therapy is the mainstay treatment for non-metastatic NPC. The prevailing use of intensity-modulated radiotherapy (IMRT) in combination with chemotherapy has substantially improved the treatment outcome in terms of local control and overall survival (OS), (1–3) and approximately 10 to 15% of the patients may suffer from locoregionally recurrence. (4) However, radiation-related toxicities become a concern for patients with NPC who underwent IMRT, especially for long-term survivors. IMRT may not sufficiently discriminate tumor volumes from critical organs at risk (OARs) in close proximity, especially for large volume tumors with skull base and/or intracranial involvement, leading to substantial acute or late toxicities including severe nasopharyngeal mucositis and injury of the central nervous system (CNS). Therefore, more precise and conformal radiation techniques are needed to further improve the toxicity profile of patients with NPC.

Particle radiation therapy (such as proton and carbon ion) delivers dose more conformally to the tumor because of its characteristic Bragg peak. (5) Our dosimetric study showed carbon-ion radiotherapy (CIRT) significantly reduces dose to surrounding organs (including brainstem and temporal lobes) for patients with recurrent nasopharyngeal carcinoma, compared to IMRT. (6) Although not investigated in newly diagnosed NPC patients, it is reasonable to speculate CIRT can provide similar improvement in dose distribution. Akbaba et al., in an initial study, showed the satisfactory efficacy and toxicity profile of IMRT with CIRT boost; however, this study was limited by the relatively small sample size of 26 patients. (7) Clearly, additional knowledge is needed for a more in-depth understanding of the merit of CIRT in the management of patients with NPC.

The purpose of this report is to document the outcome of a relatively large group of NPC patients treated at the Shanghai Proton and Heavy Ion Center (SPHIC) with mixed-beam radiotherapy using photon-based IMRT and CIRT boost.

## Materials and Methods

### Patients

Consecutive patients with newly diagnosed and histologically confirmed non-keratinizing carcinoma of the nasopharynx treated at the SPHIC according to our prospectively designed treatment protocol between June, 2015 and June, 2018 were included in this retrospective analysis. Patients with distant metastasis, previous irradiation to the head and neck region for any malignancies, or treated with a regimen other than IMRT with CIRT boost were excluded. Multidisciplinary team evaluation and discussion prior to treatment was mandatory for all patients treated at the SPHIC. Baseline workup for all patients was performed as previously described, (8) including a complete history and thorough physical examination, fiberoptic and/or indirect nasopharyngoscopy, complete blood count and serum chemistry panel, electrocardiogram, and urinalysis. Magnetic resonance imaging (MRI) of the head and neck region was required to evaluate the extension of the locoregional disease unless medically contraindicated. Whole-body positron emission tomography (PET)/computed tomography (CT) (can be replaced by chest CT, abdominal ultrasound, and bone scan) was performed for each patient to rule out distant metastasis. All patients were restaged according to the 8th edition of the American Joint Committee on Cancer (AJCC) staging classification for their disease.

### Radiation Therapy

Patients were set up in supine position with thermoplastic masks to immobilize head, neck, and shoulders. Simulation CT scan was performed at a 1.5-mm cut covering the region from the vertex to the inferior margin of the clavicular heads. MRI performed at treatment position was fused to simulation CT for delineating targets and organs at risk (OARs).

The gross tumor volume (GTV) included the primary disease located in the nasopharynx and metastatic cervical lymph nodes detected by physical examination and imaging studies (MRI, CT, and/or PET/CT). CTV boost was defined as the GTV plus a margin of 3-mm for the primary tumor (located in the nasopharynx) and 1- to 3-mm for cervical lymph nodes. High-risk clinical target volume (CTV1) was defined as the GTV plus a 5-mm margin. CTV1 should also include the entire nasopharynx, parapharyngeal space, retropharyngeal lymph nodes, inferior sphenoid sinus, skull base, clivus, pterygopalatine fossa, posterior 1/3 to 1/4 of the nasal cavity/maxillary sinuses, and bilateral nodal levels II through Va (ipsilateral nodal level I will be included when patients had positive level II nodes). A CTV2 was contoured to include nodal levels IV and Vb if the patient had ipsilateral upper neck lymphadenopathy. To account for positioning error and range uncertainty (for CIRT), planning target volumes (PTV1, PTV2, and PTV boost) were created by adding a 3- to 6-mm margin to the CTVs. PTV1 and PTV2 were treated using IMRT with a dose of 56 Gy/28 fractions and 50.4 Gy/28 fractions, respectively. A boost dose of 15–17.5 GyE in five to six fractions (2.5–3.5 GyE per fraction) was prescribed to the PTV boost using CIRT. Based on the treatment response at completion of the radiotherapy, a further boost dose could be given to the residual disease (lymph node and/or disease in the nasopharynx) at the discretion of the attending radiation oncologist. The IMRT-Carbon Ion treatment planning was integrated finally to assess the constraints of dose to OARs and to avoid potential severe toxicities caused by hypofractionated CIRT in this group of patients with relatively well prognosis; more stringent dose constraints based on the historical paper of Emami was used. (9) The treatment plans of IMRT and CIRT boost were assessed separately, and then a sum plan was generated to assess the overall dose coverage of the targets and the OAR constraints. All patients received simulation CT scan and MR scan before CIRT, and re-calculation of the dose distribution was performed on the latest simulation CT. Replan was carried out if deemed necessary.

Pencil beam scanning (PBS) technique was used for carbon-ion radiotherapy using the Siemens IONTRIS system, and planning was done using the Siemens Syngo treatment planning system versions VC 11&13. PBS planning was achieved by multi-field optimization with 2–3 fields. As for IMRT plans, nine-field technique or double/triple-arc volumetric-modulated arc therapy was used.

### Systemic Therapy

Concurrent chemotherapy was recommended for patients with locally advanced (*i.e.*, T ≥ 2) and/or node positive disease. Concurrent chemotherapy consisted of weekly or tri-weekly cisplatin or nedaplatin. Induction chemotherapy was prescribed to patients with stage III/IV NPC. The most frequently used regimens were cisplatin/nedaplatin with docetaxel (TP) or gemcitabine (GP). Adjuvant chemotherapy was generally not provided. Nimotuzumab was prescribed in place of or along with concurrent chemotherapy at the discretion of the primary oncologists for some of the patients.

### Follow-Up and Toxicity Evaluation

Patients were evaluated weekly during radiation therapy. All patients were asked to be followed up at our institution. Patients unable to follow up in person were followed locally, and results were communicated. The first follow-up was at 4 weeks after the completion of radiotherapy, then every 3 months for the first two years, every 6 months up to the fifth year, and annually thereafter. The Common Terminology Criteria for Adverse Events (CTCAE) version 4.03 was used to grade acute (within 90 days counting from the start of radiotherapy) and late (any time after 90 days from the start of radiotherapy) adverse events.

### Statistical Analysis

Treatment response was assessed according to the Response Evaluation Criteria in Solid Tumors (RECISTs) version 1.1 (10). Overall survival (OS) was defined as the duration from diagnosis to death. Progression-free survival (PFS) was defined as the duration from diagnosis to disease progression or death. Local control, regional control, and distant control were defined as durations from diagnosis to local, regional, and distant failure, respectively. Kaplan–Meier method was used to calculate the OS rate, PFS rate, local control rate, regional control rate, and distant control rate. Although multivariable analysis was not performed due to the limited events that occurred in the study cohort, log-rank test was used to examine the associations between T category and local control, N category and distant control, and response to induction chemotherapy and PFS. P-values <0.05 were considered as statistically significant. All analyses were performed with R statistical software (version3.5.1; R Foundation, Austria).

## Results

### Baseline and Treatment Characteristics

**Figure 1:**
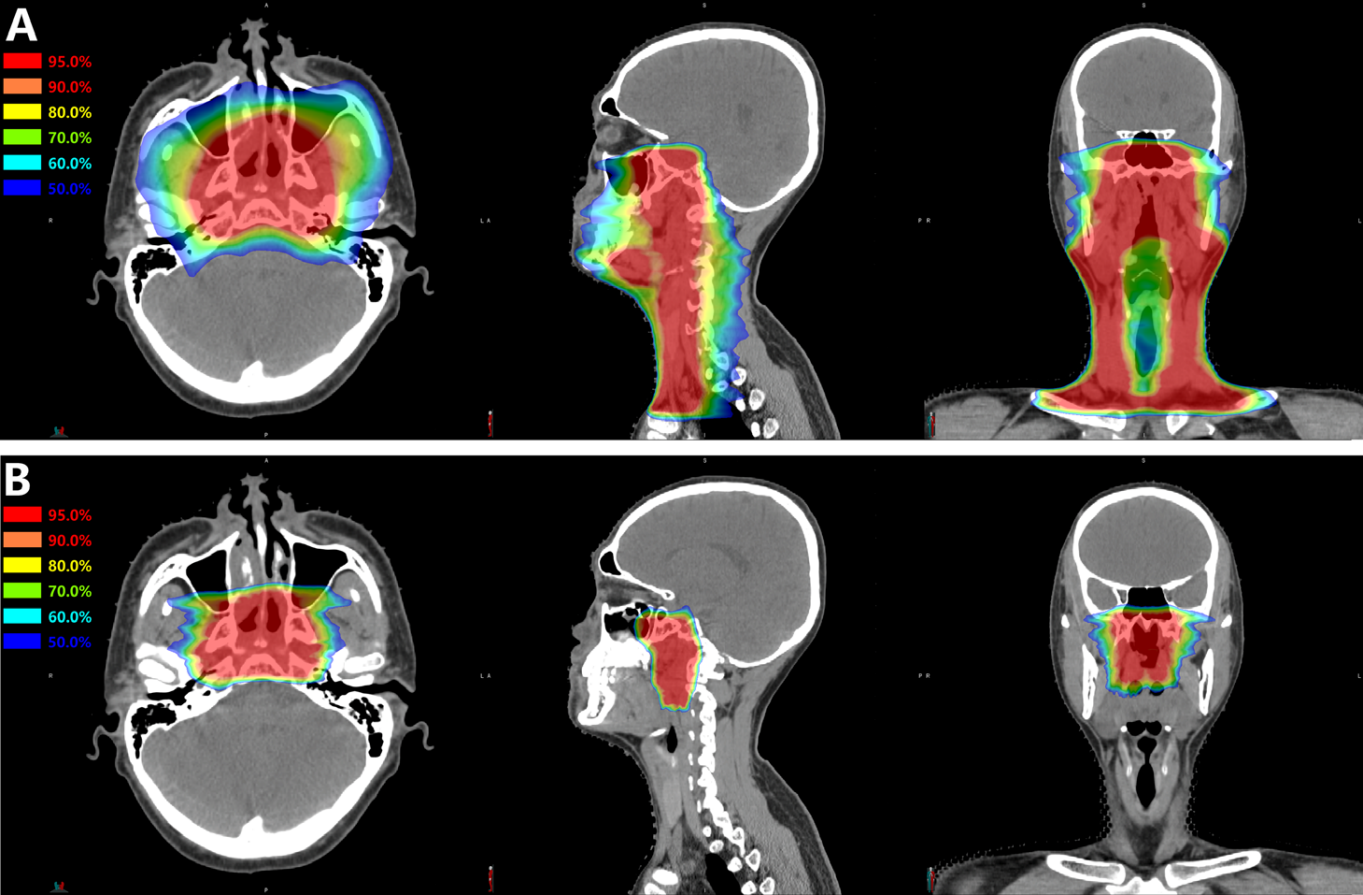
A typical treatment plan of mixed **(A)** IMRT plus **(B)** CIRT boost for nasopharyngeal carcinoma. This was a patient with T3N3M0 disease treated with 56 Gy of IMRT in 28 fractions plus 15 GyE of CIRT in 5 fractions.

Ninety-one consecutive patients with newly diagnosed and non-metastatic nasopharyngeal carcinoma were treated at the SPHIC between June, 2015 and June, 2018. Among those, 69 patients treated according to our prospectively designed protocol of IMRT plus CIRT boost were enrolled in this retrospective analysis. The remaining 22 patients who received other treatment regimens (such as proton therapy) were excluded from the current study. All patients received 56 Gy/28 fractions to the PTV1, and 39 patients received 50.4 Gy/28 fractions to the PTV2. The median boost dose of CIRT was 15.0 GyE (range, 15.0–17.5 GyE), and the median fraction size was 3 GyE (range, 2.5–3.5GyE) per fraction (**Figure 1**). According to the response evaluation at completion of the radiotherapy, two patients received further boost to the lymph nodes using IMRT (6.6 Gy/3 fractions and 6 Gy/3 fractions, respectively); nine patients received further boost using CIRT to nasopharynx or lymph nodes with a median dose of 6 GyE (range, 3–8 GyE) in one to three fractions. The baseline characteristics and treatment modalities of the 69 patients included in this analysis were detailed in **Table 1**.

**Table 1:** Characteristics at baseline and treatment modalities.

Characteristics	No. of patients (%)
Age	
Median (range)–year	48 (14–68)
<60 years	54 (78.26%)
≥60 years	15 (21.74%)
Gender	
Female	13 (18.84%)
Male	56 (81.16%)
Tumor category	
T1	18 (26.09%)
T2	10 (14.49%)
T3	29 (42.03%)
T4	12 (17.39%)
Node category	
N0	5 (7.25%)
N1	30 (43.48%)
N2	22 (31.88%)
N3	12 (17.39%)
Disease stage	
I	1 (1.45%)
II	17 (24.64%)
III	29 (42.03%)
IV	22 (31.88%)
Histology	
Non-keratinizing carcinoma, differentiated type	9 (13.04%)
Non-keratinizing carcinoma, undifferentiated type	60 (86.96%)
Induction Chemotherapy	
with	59 (85.51%)
without	10 (14.49%)
Concurrent chemotherapy	
with	60 (86.96%)
without	9 (13.04%)

GTV, gross tumor volume; CTV, clinical target volume; GyE, gray equivalent.

### Disease Control and Survival

**Figure 2:**
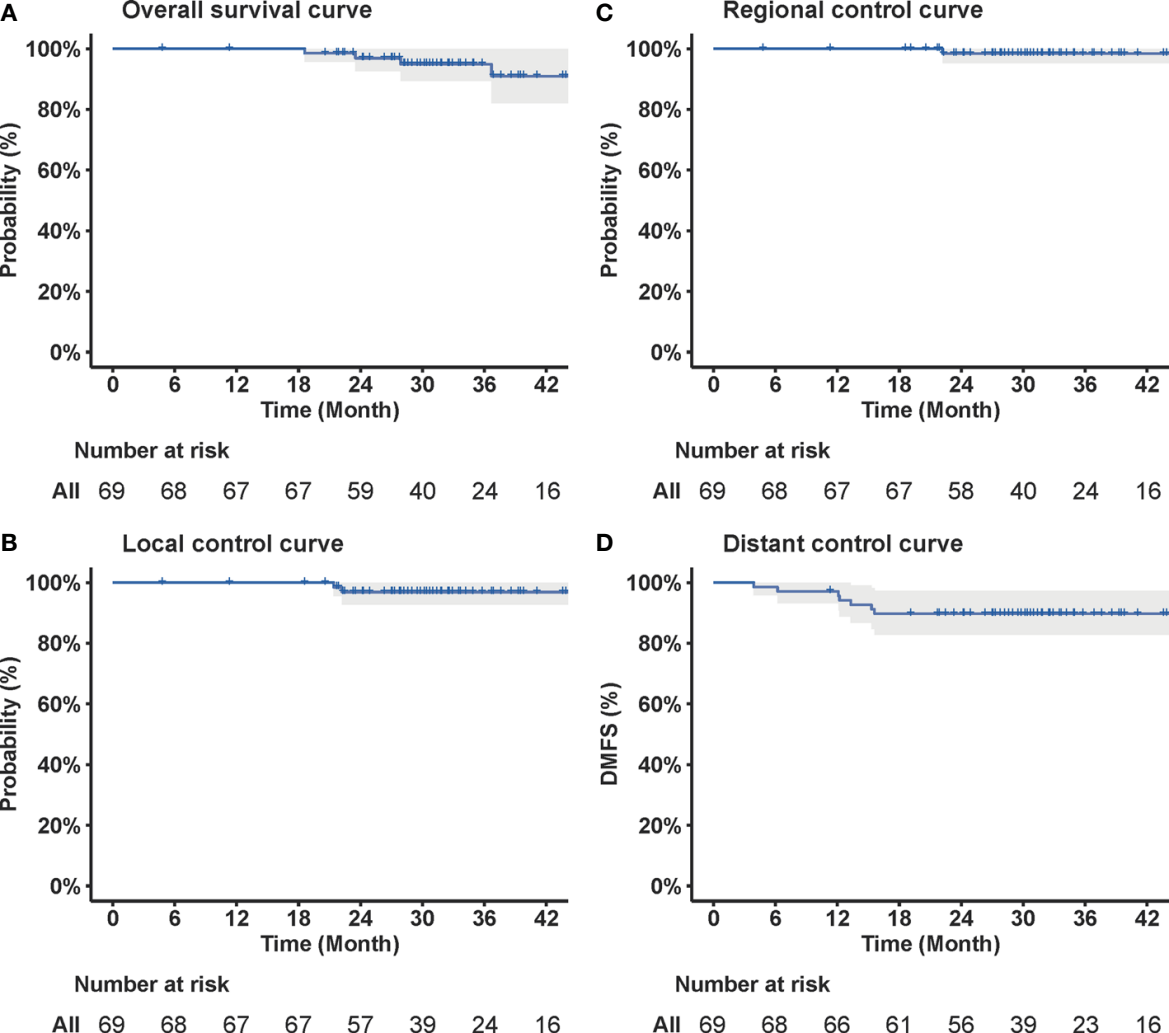
Curves of **(A)** overall survival, **(B)** local control, **(C)** regional control, and **(D)** distant control of the 69 patients with newly diagnosed nasopharyngeal carcinoma included in the current study. The corresponding OS, local control, regional control, and distant control rates at 3-year were 94.9, 96.9, 98.4, and 89.7%, respectively.

At a median follow-up time of 31.8 (range, 4.8–62.3) months, four patients died (all died of distant metastasis), and three, one, and seven patients developed local, regional, and distant failure, respectively. All of the three patients developed local recurrence had locally advanced (T4) disease. All of the seven patients who developed distant metastasis had node-positive disease, and one, three, and three patients had N1, N2, and N3 disease, respectively. The 3-year OS, PFS, local control, regional control and distant control rates were 94.9% [95% confidence interval (CI), 89.4–100.0%), 85.2% (95% CI, 77.2–94.1%), 96.9% (95% CI, 92.7–100.0%), 98.4% (95% CI, 95.3–100.0%), and 89.7% (95% CI, 82.8–97.2%), respectively (**Figure 2**).

**Figure 3:**
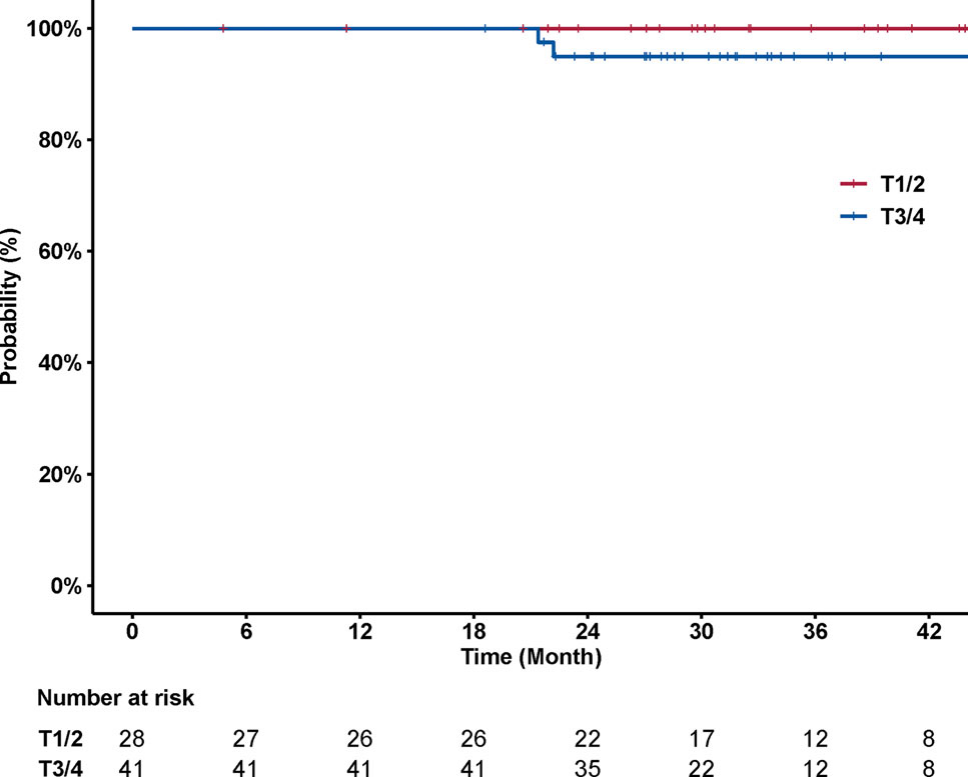
Curves of local control stratified by T category (T1/2 *versus* T3/4). No significant association was observed between local control and T category (*p* = 0.157). The 3-year local control rates were 100% [95% confidence interval (CI), 100–100%] and 94.9% (88.3–100%) for T1/2 *versus* T3/4 disease, respectively.

**Figure 4:**
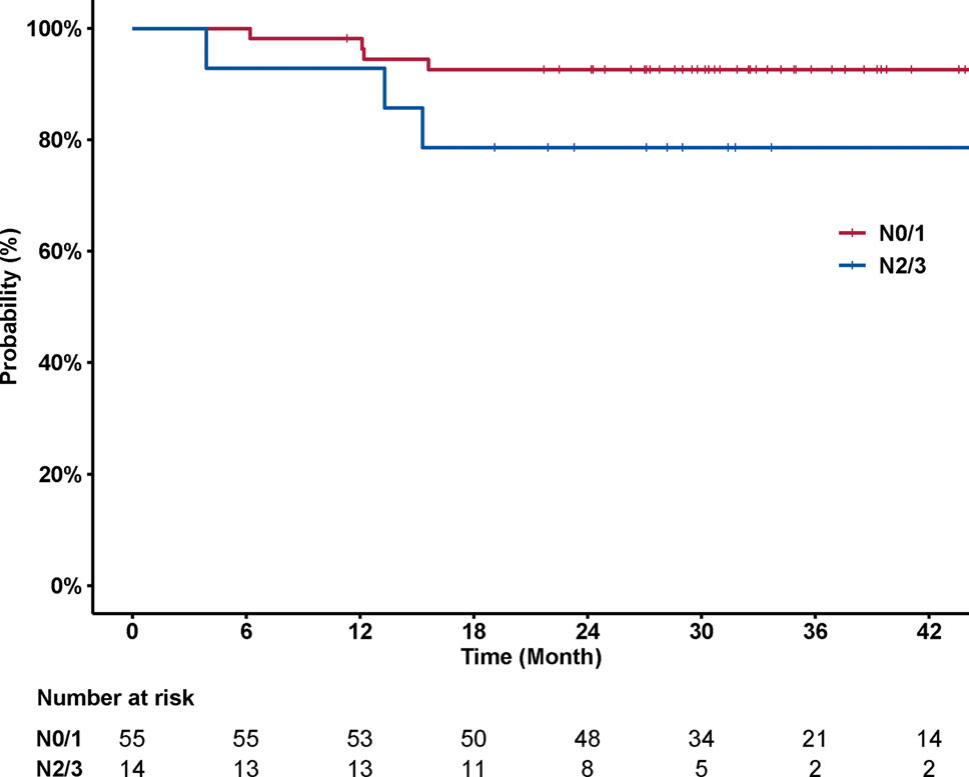
Curves of distant control stratified by N category (N0/1 *versus* N2/3). N2/3 disease was associated with significantly increased risk of developing distant metastasis (*p* = 0.041). The 3-year distant control rates were 82.0% (95% CI, 70.0–96.1%) for patients with N2/3 disease and 97.1% (95% CI, 91.8–100%) for patients with N0/1/2 disease, respectively.

No significant association was observed between disease control and T category (*p* = 0.157) (**Figure 3**). The 3-year local control rates were 100% (95% CI, 100–100%) and 94.9% (95% CI, 88.3–100%) for T1/2 *versus* T3/4 disease, respectively. The association between nodal status and incidence of distant metastasis was also examined. Patients with N2/3 disease had a significantly higher chance of developing distant metastasis, compared to patients with N0/1 disease (*p* = 0.041) (**Figure 4**). The corresponding 3-year distant control rates were 82.0% (95% CI, 70.0–96.1%) for patients with N2/3 disease and 97.1% (95% CI, 91.8–100%) for patients with N0/1 disease.

**Figure 5:**
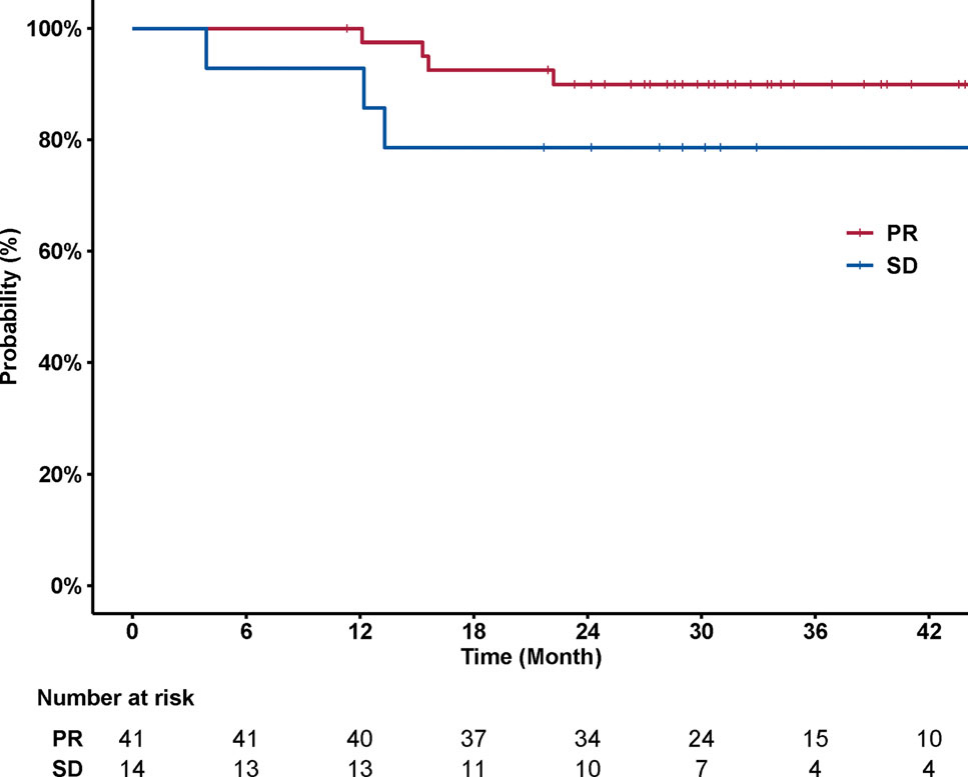
Curves of PFS stratified by response to induction chemotherapy (PR *versus* SD). No significant association was observed between PFS and response to induction chemotherapy (*p* = 0.097). The 3-year PFS rates were 89.9% (95% CI, 81.0–99.8%) *versus* 78.6% (95% CI, 59.8–100%) for patients who achieved or did not achieve PR, respectively.

A total of 59 patients in the current cohort received induction chemotherapy. Response to induction chemotherapy was evaluated for 55 patients whose baseline MRI images were available. Partial response (PR) was achieved in 41 patients (74.5%), and the remaining 14 patients (25.5%) had stable disease. Univariable analysis showed no significant association between response to induction chemotherapy (PR *versus* SD) and disease control. The 3-year PFS rates were 89.9% (95% CI, 81.0–99.8%) *versus* 78.6% (95% CI, 59.8–100%) for patients who achieved PR or not (*p* = 0.097) (**Figure 5**).

### Acute and Late Toxicities

Mixed treatment of IMRT with CIRT boost was well tolerated. Acute adverse events that occurred during radiotherapy were detailed in **Table 2**. Severe (defined as ≥grade 3) acute toxicities induced by radiation therapy were observed in two patients (dermatitis). More common severe acute hematological toxicities most likely associated with chemotherapy included neutropenia, thrombocytopenia, leucopenia, and anemia. Grade 1 or 2 radiation-induced late toxicities included dysgeusia in 22 (31.8%) patients, xerostomia in 59 (85.5%) patients, and hearing impairment in 11 (15.9%) patients. No severe radiation-induced late toxicity was observed at the time of analysis.

**Table 2:** Acute adverse events that occurred during treatment.

Toxicities	None	Grade 1	Grade 2	Grade 3	Grade 4
Mucositis	2 (2.9%)	40 (58.0%)	27 (39.1%)	0	0
Dermatitis	4 (5.8%)	55 (79.7%)	8 (11.6%)	2 (2.9%)	0
Xerostomia	7 (10.1%)	37 (53.6%)	25 (36.2%)	0	0
Dysgeusia	35 (50.7%)	31 (44.9%)	3 (4.3%)	0	0
Nausea and vomiting	21 (30.4%)	34 (49.3%)	14 (20.3%)	0	0
Hearing impairment	59 (85.5%)	10 (14.5%)	0	0	0
Neutropenia	21 (30.4%)	15 (21.7%)	23 (33.3%)	8 (11.6%)	2 (2.9%)
Leucopenia	7 (10.1%)	17 (24.6%)	27 (39.1%)	18 (26.1%)	0
Anemia	10 (14.5%)	32 (46.4%)	24 (34.8%)	3 (4.3%)	0
Thrombocytopenia	41 (59.4%)	9 (13.0%)	12 (17.4%)	7 (10.1%)	0

## Discussion

This is the largest study assessing the efficacy and toxicity profile of mixed IMRT plus CIRT boost for patients with NPC. A total of 69 patients were included in the current study. Among all patients, 73.9% of the patients had locoregionally advanced (stage III/IV) disease and 49.3% of the patients had N2/3 lymphadenopathy, representing a cohort with unfavorable prognostic characteristics. Our results showed that the mixed radiation strategy could provide a promising disease control and was associated with only mild toxicities. The 3-year OS, PFS, local control, regional control, and distant control rates were 94.9, 85.2, 96.9, 98.4, and 89.7%, respectively. The acute toxicities related to IMRT plus CIRT boost were mild, and severe dermatitis was observed in only two patients. Severe radiation-induced late toxicities were not observed at the time of analysis. Univariable analysis showed that advanced cervical lymphadenopathy (N2/3) was associated with significantly worse distant control (*p* = 0.041).

The current standard-of-care for NPC is IMRT with or without chemotherapy according to disease stage of the patients. (11) After IMRT-based comprehensive treatment, satisfactory local and regional control could usually be achieved. In a study of 749 NPC patients treated by IMRT with or without chemotherapy, the authors showed a 5-year OS and local recurrence free survival (LRFS) rates of 82 and 94.6%, respectively (12). Patients with advanced stages had significantly worse disease control and OS. The 5-year OS rates for patients with stages I–IV were 97.4, 93.8, 81.8, and 69.7%, respectively. While IMRT-based treatment achieves satisfactory outcomes in terms of in-field disease control for NPC patients, critical organs in close proximity may receive a substantial radiation dose, especially for those with locally advanced disease. Particle beam radiotherapy (PBRT) is of great interest to further improve the therapeutic ratio. Accelerated charged particle beams (such as proton beam and carbon-ion beam) are able to deliver dose more conformally to the tumor target due to their physical advantages (5), thus potentially reducing the radiation-induced toxicities.

Results of pilot studies have showed PBRT could provide promising outcomes. Lewis et al. demonstrated that intensity-modulated proton therapy (IMPT) could provide dosimetric advantages for NPC patients with lower dose delivered to the surrounding normal tissues as compared to IMRT (13). In this study of nine patients, the 2-year locoregional control, DMFS, and OS rates after IMPT were 100, 88.9, and 88.9%, respectively. The relatively low DMFS and OS rates were at least partially due to the unfavorable nodal status of the study cohort (89% of the patients had neck lymphadenopathy and 56% had N2 disease). In terms of acute toxicities, four and one patients developed grade 3 dermatitis and mucositis, and all mucositis occurred within the treatment field. No patient developed grade 3 or above late toxicities. Alterio et al. retrospectively compared the efficacy of IMRT and IMRT with proton beam boost in patients with locally advanced NPC (17 patients received IMRT alone and 27 patients received IMRT with proton therapy boost) (14). Although no significant difference was detected in the univariable analysis of local progression-free survival (89 *vs* 94%) and PFS (69 *vs* 76%) at 2 years, mixed treatment of IMRT with proton boost was associated with significantly reduced severe acute toxicities (11 *vs* 76%, p < 0.001).

Knowledge of CIRT for patients with NPC is scarce. Akbaba et al., in an initial study of 26 patients, examined the effectiveness and safety of IMRT with CIRT boost either as definitive or postoperative treatment for NPC (7). Although most of the patients had locally advanced disease, the 2-year OS, local control, and DMFS rates after bimodal treatment with IMRT and CIRT boost were 100, 95, and 93%, respectively. Till the latest follow-up, severe acute and late toxicities were observed in 20 and 16% of the patients. However, this study was limited by its relatively small sample size. In addition, surgery, which is not conventional in the management of NPC and may introduce bias in interpreting the efficacy of radiotherapy, was used in 35% of the patients. Our study showed that mixed treatment of IMRT with CIRT boost could provide satisfactory OS and local control comparable to historic results of IMRT (15), even though more patients in the current cohort had locoregionally advanced disease. Meanwhile, due to its more conformal dose distribution, mixed treatment of IMRT with CIRT boost could provide better protection for the surrounding organs. Mucositis is one of the most common acute toxicities during the treatment for NPC patients, and the incidence of severe mucositis (grade 3/4) ranges between 20 and 40% in patients treated by IMRT (16–18). In our study, grade ≥3 mucositis was not observed. Although use of different evaluation criteria (such as RTOG/EORTC *vs* CTCAE) might introduce certain inconsistency in toxicity grading, CIRT could substantially reduce the incidence of severe mucositis considering no patient in the current cohort required PEG-tube feeding. Of note, in the study conducted by Akbaba, 24% of the patients required tube feeding, despite the similar radiotherapy technique used for treatment (7). The underlying for such discrepancy is difficult to confirm but could be due to the difference in target volume delineation between the two centers. Late toxicities of the current study cohort were mild and acceptable. Xerostomia was the most common late toxicity and was observed in 85.5% of the patients (none had grade ≥3) in the current study. Although the incidence of xerostomia was comparable in our study and in patients treated by IMRT alone, none of our patients developed moderate to severe toxicities otherwise. Neurologic injuries (such as temporal lobe necrosis and cranial neuropathy) could significantly reduce the quality of life of the patients. The reported incidence of neurologic injuries after photon based IMRT ranges between 5 and 20% depending on the follow-up time of the study cohorts. (18–20) In an analysis of 208 NPC patients, 16 (7.5%) patients developed neurologic complications at a median follow-up of 78 months, and more than half of the events occurred within the first 3 years. (19) In comparison, no obvious neurologic injuries were detected in the current study at a median follow-up time of 31.8 months.

CIRT is expensive and its availability is limited. To take better advantage of the CIRT, it is helpful to identify the most suitable candidates. In addition to its conformal dose distribution, the higher relative biological effects of the CIRT enable more effective killing of the resistant tumor cells. Although non-keratinizing NPC in endemic regions is considered radiosensitive, 10–15% of the patients may still develop local recurrence after radiotherapy-based comprehensive treatment, partially due to the tumor heterogeneity. Those patients with relatively resistant tumors may benefit from CIRT the most. Therefore, markers for radiosensitivity to CIRT are in need. The prognostic/predictive value of previously established markers for IMRT, such as EBV-DNA level, response to induction chemotherapy, and T category, should be re-evaluated for CIRT.

To the best of our knowledge, this is the largest study examining the role of CIRT in the treatment for NPC; however, a median follow-up time of 31.8 months was relatively short. Although longer follow-up is necessary to evaluate the late toxicities more accurately, our results have revealed that at a median follow-up time of 31 months, the toxicity profile related to IMRT with CIRT boost was mild and acceptable, and no neurologic events occurred; however, because of the potentially confounding factors and relatively short follow-up time, the question, whether addition of CIRT could significantly reduce the radiation-induced toxicities compared to IMRT alone, should be answered in a well-designed matched study or a prospective clinical trial. In addition, because of the limited events that occurred during the follow-up and the retrospective nature of the current study, the results of the univariable analysis could be biased by the potential confounding factors.

## Conclusion

In conclusion, IMRT combined with CIRT boost provides comparable disease control and a favorable toxicity profile for patients with NPC, as compared to historic studies. Further follow-up is necessary to evaluate the long-term disease control and toxicities more accurately. Randomized trials or matched studies are necessary to more accurately address whether addition of particle therapy can further improve the disease control and survival for NPC patients. Currently, a randomized phase II study to compare IMRT and proton beam therapy, both followed by CIRT boost, is ongoing at our center.

## Data Availability Statement

The raw data supporting the conclusions of this article will be made available by the authors, without undue reservation.

## Ethics Statement

The studies involving human participants were reviewed and approved by Institutional review board of Shanghai Proton and Heavy Ion Center. Written informed consent to participate in this study was provided by the participants’ legal guardian/next of kin.

## Author Contributions

JH: Conceptualization, data curation, formal analysis, methodology, validation, visualization, funding acquisition, writing-original draft, and writing-review and editing. QH: Data curation, formal analysis, methodology, validation, visualization, writing-original draft, and writing-review and editing. JG: Data curation, validation, writing-original draft, and writing-review and editing. WH: Data curation, validation, writing-original draft, and writing-review and editing. JY: Data curation, validation, writing-original draft, and writing-review and editing. XG: Data curation, validation, writing-original draft, and writing-review and editing. XQ: Data curation, validation, writing-original draft, and writing-review and editing. LK: Conceptualization, formal analysis, methodology, validation, resources, funding acquisition, supervision, writing-original draft, and writing-review and editing. JL: Conceptualization, formal analysis, methodology, validation, resources, funding acquisition, supervision, writing-original draft, and writing-review and editing. All authors contributed to the article and approved the submitted version.

## Funding

This study was supported by the National Key Research and Development Program of China (project no. 2018YFC0115700); Shanghai Rising-Star Program (project no. 19QB1405500); and Clinical Research Plan of Shanghai Shenkang Hospital Development Center (Project No. SHDC12017X13).

## Conflict of Interest

The authors declare that the research was conducted in the absence of any commercial or financial relationships that could be construed as a potential conflict of interest.

## Publisher’s Note

All claims expressed in this article are solely those of the authors and do not necessarily represent those of their affiliated organizations, or those of the publisher, the editors and the reviewers. Any product that may be evaluated in this article, or claim that may be made by its manufacturer, is not guaranteed or endorsed by the publisher.
